# NBL1 Reduces Corneal Fibrosis and Scar Formation after Wounding

**DOI:** 10.3390/biom13111570

**Published:** 2023-10-24

**Authors:** Chi-Hao Tsai, Emily Liu, Andrew Phan, Krystal Lynn Lu, Hua Mei

**Affiliations:** 1 Department of Ophthalmology, School of Medicine, The University of North Carolina at Chapel Hill, Chapel Hill, NC 27599, USA; 2 Department of Psychology and Neuroscience, School of Medicine, The University of North Carolina at Chapel Hill, Chapel Hill, NC 27599, USA; 3 Department of Cell Biology and Physiology, School of Medicine, The University of North Carolina at Chapel Hill, Chapel Hill, NC 27599, USA

**Keywords:** NBL1, corneal scar, fibrosis, corneal wound

## Abstract

Corneal scarring is a leading cause of blindness. Currently, there is no treatment to prevent and/or reduce corneal scar formation under pathological conditions. Our previous data showed that the NBL1 protein, also termed the DAN Family BMP (Bone morphogenetic protein) Antagonist, was highly expressed in corneal stromal cells upon wounding. Here, we examined the function of NBL1 in corneal wound healing. Mouse corneas were mechanically wounded, followed by a 2-week treatment using NBL1. Wounded corneas treated with vehicle or an Fc tag served as controls. Compared with the controls, NBL1 treatment facilitated wound re-epithelialization, partially restored the stromal thickness, and significantly reduced corneal scar formation. NBL1 treatment did not decrease immune cell infiltration, indicating that the anti-scarring effect was not dependent on immune suppression. We further examined the anti-fibrotic effect of NBL1 on human corneas. Pairs of human corneas were induced to form myofibroblasts (a key player in fibrosis and scarring) upon wounding and incubation in a medium containing TGF-β1. The OS corneas were treated with Fc as a control, and the OD corneas were treated with NBL1. Compared with the control, human corneas treated with NBL1 had significantly fewer myofibroblasts, which was consistent with these mouse data. A further study revealed that NBL1 treatment inhibited BMP canonical (phospho-Smad1/5) and no-canonical (phospho-p38) pathways in human corneas. Data show that NBL1 reduced corneal fibrosis and scar formation in mice and cultured human corneas. The underlying molecular mechanism is not certain because both anti-fibrotic Smad1/5 and pro-fibrotic p38 pathways were inhibited upon NBL1 treatment. Whether the p38 pathway dominates the Smad1/5 pathway during corneal fibrosis, leading to the anti-fibrotic effect of NBL1, needs further investigation.

## 1. Introduction

Corneal scarring is a major cause of blindness worldwide [1,2,3,4]. Common causes of corneal scars include injury/trauma, infections, and secondary outcomes of other diseases such as keratoconus, severe dry eye, and diabetic keratopathy. The current treatment protocol is to control the cause of the corneal wound (e.g., remove the foreign bodies, treat infection, or control the primary disease) and to provide eye patches, topical antibiotics, and analgesics to help the cornea heal by itself. There is no drug approved by the FDA to prevent or reduce corneal scar formation during the healing process. One clinical approach to minimize corneal scarring after trauma is to reduce inflammation with the topical application of corticosteroids. However, the anti-scarring effect of this therapy is controversial. Some clinical trials showed that topical application of corticosteroids had no beneficial or harmful effect on rescuing the best-corrected visual acuity [5,6]. In Nocardia-related ulcers, corticosteroid use may even lead to larger scars, probably due to recurrent infections and delayed epithelial wound closure [6,7]. Corneal scarring is also one of the major and severe complications after ocular surgeries, especially for surface ablation procedures, including photorefractive keratectomy (PRK), epi-LASIK, and phototherapeutic keratectomy (PTK). To reduce corneal scar formation, surgeons use mitocycin-C (MMC) to treat the eye surface after the procedure. MMC is a chemotherapeutic agent that is a potent DNA crosslinker, inhibits protein synthesis, and prevents cell proliferation. Although MMC showed benefits in minimizing corneal scar/haze formation [8,9], probably by inhibiting the proliferation of myofibroblast precursor cells, it also inhibits protein synthesis and the proliferation of normal corneal cells, which raises concerns about its long-term safety, especially for stem cells that are responsible for the long-term cell regeneration and are susceptible to DNA damage. Due to the lack of effective drugs able to prevent or reduce corneal scar formation after trauma and ocular surgeries, patients may suffer from corneal scarring and vision loss, which impairs life quality and self-sustainability. New therapeutic approaches to prevent or reduce corneal scars have been a research focus [10,11,12]. 

Based on our previous single-cell RNA sequencing (scRNAseq) data [13], during scarless healing of a wound restricted to the epithelial layer, corneal stromal cells were activated and expressed a high level of NBL1, a secreted protein known as a BMP antagonist, leading to the hypothesis that NBL1 might play a role in corneal wound healing. In this study, we examined this hypothesis using an in vivo mouse model and an ex vivo human organ culture model. 

## 2. Materials and Methods

### 2.1. Animal

C57BL/6J mice (The Jackson Laboratory) at around 2 months old with an approximately equal number of male and female mice were used in this study. The mice were bred and housed in standard cages and fed water and chow ad libitum. The animal experiments were conducted under the protocol approved by the Institutional Animal Care and Use Committee (IACUC) at the University of North Carolina at Chapel Hill and were in compliance with the Association for Research in Vision and Ophthalmology Statement for the Use of Animals in Ophthalmic and Vision Research. 

### 2.2. Reagents

Recombinant human DAN Fc Chimera (NBL1) and recombinant human IgG Fc were purchased from R&D Systems (Minneapolis, MN, USA). Fluorescein sodium and other reagents used in molecular analysis were purchased from Sigma (St. Louis, MO, USA) unless otherwise stated.

### 2.3. Assessing the Bioactivity of the Purchased NBL1 Protein in Mouse and Human Cells

The 3T3-J2 and HEK293 cell lines were obtained from the American Type Culture Collection (Manassas, VA, USA). Both cells were maintained in Dulbecco’s modified Eagle’s medium (DMEM, 11995-123; Gibco, Grand Island, NY, USA), supplemented with 10% heat-inactivated fetal bovine serum, 10,000 U/mL penicillin, 10 mg/mL streptomycin, and 25 µg/mL amphotericin B (ThermoFisher Scientific, Waltham, MA, USA) in a humidified incubator with 5% CO_2_ at 37 °C. The cells were seeded at 3 × 10^4^ cells/well in a 4-well chamber slide overnight, followed by serum starvation for 1 h. Then, the cells were treated with control (“Ctl,” vehicle only), BMP4 (50 ng/mL, R&D Systems, 314-BPE), BMP4 + 1× NBL1 (3 µg/mL), BMP + 1 × Fc (same molar concentration as 1 × NBL1), BMP4 + 10 × NBL1 (30 µg/mL), and BMP + 10 × Fc (same molar concentration as 10 × NBL1) for 4 h before examination using immunocytochemistry (ICC).

### 2.4. Immunocytochemistry (ICC)

The cells were fixed in 2% paraformaldehyde for 5 min, washed with PBS 4 times, and permeabilized with 0.1% Triton–X100 for 5 min. Then, the cells were blocked in 2% BSA for 30 min and incubated in the primary antibody (anti-p-Smad1/5, Cell Signaling Technology, Danvers, MA, USA) in 2% BSA at 4 °C overnight, followed by the detection of the signals using the secondary antibody (Alexa Fluor 488, ThermoFisher Scientific). The fluorescent signals were observed under the fluorescent microscope (Olympus IX-71, Tokyo, Japan).

### 2.5. Mouse Wound Healing Procedure

The central corneal epithelium and the anterior stroma of the mice were removed using the Algerbrush II corneal rust ring remover (Alger Co, Lago Vista, TX, USA). After practice, we managed to remove consistently around 25–35% of the corneal surface (examined by fluorescein staining, Millipore Sigma, Burlington, MA, USA) with a depth of 50–60% of corneal stromal thickness (examined by optical coherence tomography (OCT), MICRON IV, Phoenix Technology Group, Tempe, AZ, USA). The occasional outliers were excluded from the experiments. Wounded mouse corneas were treated with NBL1 (“1×”: 3 µg/mL, 1 µL/treatment; “10×”: 30 µg/mL, 1 µL/treatment) using subconjunctival injections (injected every 3 days) for 2 weeks. Treatment with Fc protein at corresponding molar concentrations (“1 × Fc” and “10 × Fc”) served as control groups. Treatment with the vehicle served as an additional control (“Ctl”). The mice were administered with neomycin and polymyxin B sulfate ophthalmic ointment (Bausch + Lomb, Bridgewater, NJ, USA) after each injection to avoid infection. The mouse eyes were observed under a Leica MZ6 stereomicroscope (Leica, Wetzlar, Germany) during the course and at the end of the treatment to observe the corneal re-epithelization and scar formation.

### 2.6. Examination of the Wound Re-Epithelialization

The corneal unepithelialized wound was stained with 1% fluorescein and observed using a Leica MZ6 stereomicroscope. The wound size was calculated as a percentage of the corneal area using ImageJ software (Version 1.53t). 

### 2.7. Optical Coherence Tomography (OCT)

After anesthesia (ketamine 90 mg/kg and xylazine 10 mg/kg), mouse eyes were treated with a drop of 2% Methocel^®^ (OmniVision, Zurich, Switzerland). The corneal surface was observed using the MICRON IV. SD-OCT images were scanned vertically or horizontally at the indicated positions. The thickness of the corneal layers was identified and analyzed using the InSight software. 

### 2.8. Evaluation of Corneal Scar via Stereomicroscopy

The corneal scar was examined using a Leica MZ6 stereomicroscope on the dissected whole globes and dissected mouse corneas. Quantitative analysis of the corneal scar was based on a double-blind observation and grading of the dissected corneas placed on a printed number “1” by 3 independent researchers. Corneal scarring was graded as 1/clear, 2/slightly cloudy, 3/very cloudy, and 4/opaque. 

### 2.9. Evaluation of Corneal Scar and Inflammation via IHC

The 8 µm-thick cryosections of the mouse eyes were fixed in 2% paraformaldehyde, washed with PBS 4 times, and permeabilized with 0.2% Triton–X100 for 10 min. The sections were blocked in 5% BSA for 30 min and incubated in the primary antibody (anti-α-SMA, Santa Cruz Biotechnology, Dallas, TX, USA; anti-fibronectin, Invitrogen, Carlsbad, CA, USA; anti-NIMP-14, Santa Cruz Biotechnology, Dallas, TX, USA; anti-F4/80, Abcam, Cambridge, UK) in 5% BSA at 4 °C overnight, followed by the detection of signals using fluorescence-conjugated secondary antibodies. The fluorescent signals were detected using a fluorescent microscope (Olympus IX-71). The percentages of the marker-positive areas were calculated as the marker-positive area divided by the corneal stromal area around the wounding site using ImageJ software. The expression level in the no primary antibody control served as the gating value to determine the marker-positive areas. A total of 3–4 sections per mouse sample were measured, and 4–8 mice were included in each study group.

### 2.10. Organ Culture of Human Corneas

Human corneas were a generous gift from the eye bank “Miracles In Sight” (a non-profit eye bank). The experiments were performed following published protocols [14,15] with minor modifications. In brief, pairs of human corneas were obtained from Eye Bank (Miracles In Sight) with the following criteria: (1) no corneal defect and (2) death to delivery was within 4 days. The corneas were gently cleaned by removing blood residues, iris, and extra tenon and wounded by puncturing the central cornea using a 4 mm trephine to 90–100% depth (the central corneal button was almost cut through but remained in position during culture). The wounded corneas were cultured at the air–liquid interface (top of the corneal dome at the air-liquid interface) in DMEM medium supplemented with penicillin–streptomycin, gentamicin/amphotericin B, and 1 ng/mL TGF-β1 for 1 week. Both trephine cut and TGF-β1 treatment facilitated tissue fibrosis and scar formation. The experiment was conducted on 4 pairs of human corneas, of which the OS eyes were treated with NBL1 and the OD eyes were treated with Fc. The final concentration of NBL1 used in the medium was 0.3 µg/mL; the culture medium was refreshed every 2 days. At the end of the 1-week treatment, the cultured corneas were collected for cryosectioning. The IHC protocol followed the protocol mentioned above on mouse cryosections using different primary (anti-α-SMA, Santa Cruz Biotechnology; anti-p-Smad 1/5, Cell Signaling Technology; anti-p-p38, Cell Signaling Technology) and corresponding secondary antibodies. 

### 2.11. Statistical Analysis

All data are expressed as mean ± standard error. We used a *t*-test for a two-group comparison and one-way ANOVA for three- or more groups comparison with one variable to determine the statistically significant differences.

## 3. Results

**Figure 1:**
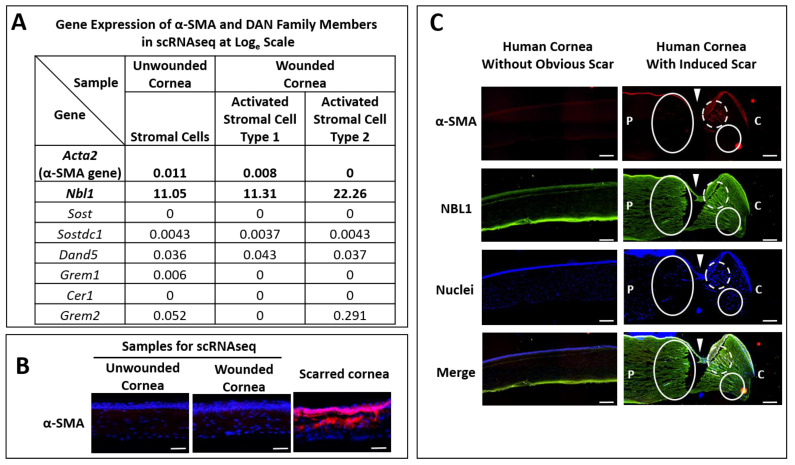
Corneal stromal cells secreted a higher level of NBL1 upon wounding compared with the unwounded status in mouse and human corneas. Previously, we established a non-scarring corneal wound healing model in mice, in which the wound was restricted to the epithelial layer and healed without scar, evidenced by the minimal expression of Acta2 (α-SMA gene) at mRNA level (**A**) and protein level (**B**) in wounded corneas compared with that in unwounded corneas. By analyzing the stromal cells using scRNAseq, NBL1 was one of the most differentially expressed genes, which showed a higher expression in activated stromal cells type 1 and type 2 compared with the quiescent stromal cells without wounding (**A**). The rest of the DAN family members showed minimal expression in corneal stromal cells with or without wounding (**A**). Similarly, in human corneas, NBL1 was highly secreted in wounded corneal stroma compared with that in unwounded stroma (**C**). Upon wounding, both activated corneal stromal cells and myofibroblasts expressed a high level of NBL1 (**C**). White arrowheads point to the wound cut by trephine. White circles indicate the activated stromal cells expressing a high level of NBL1. White dashed circles indicate the myofibroblasts expressing a high level of NBL1. P: peripheral corneal side. (**C**): central corneal side. Scale bar in (**B**): 50 µm. Scale bar in (**C**): 200 µm.

Previously, we performed a scRNAseq analysis on peripheral corneal and limbal epithelial and anterior stromal cells in mouse corneas undergoing epithelial wounding [13]. The wound was restricted to the epithelial layer with minimal damage to the stroma [13], leading to scarless wound healing, which was confirmed by the low mRNA (Figure 1A) and protein levels (Figure 1B) of α-smooth muscle actin (α-SMA, myofibroblast, and fibrotic marker) in wounded corneas, which were comparable to those from the unwounded corneas. To explore the secreted signals from corneal stromal cells during scarless wound healing, we screened out the genes from these scRNAseq data that showed a higher expression in both types (approximately equal amount in cell numbers) of activated stromal cells in wounded corneas than the stromal cells in unwounded corneas. The activated genes were ranked in a list from high to low in fold changes, and NBL1 was one of the top genes in the list. As shown in Figure 1A, NBL1 increased slightly in activated stromal cell type 1 (from 11.05 to 11.31 on the Loge scale) and increased significantly in activated stromal cell type 2 (from 11.05 to 22.26 on the Loge scale) compared with that in quiescent stromal cells in unwounded corneas. As NBL1 is a member of the DAN (Differential screening-selected gene Aberrative in Neuroblastoma) family of BMP antagonists, the other members of the DAN family were examined in these scRNAseq data and showed minimal expressions in unwounded and wounded corneas in mice (Figure 1A), suggesting that NBL1 is a major player in DAN family in cornea. To further explore whether NBL1 was secreted in human corneas upon wounding, we employed a human cornea organ culture model [14,15] in which corneas were wounded with a 4 mm trephine and incubated in a medium containing 1 ng/mL TGF-β1 to induce scarring wound healing. We observed that wound-activated human corneas expressed a significantly higher level of NBL1 compared with the unwounded human corneas, especially around the wound area (Figure 1C). Both activated stromal cells (white circle in Figure 1C) and myofibroblasts (white dashed circle in Figure 1C) expressed a high level of NBL1, suggesting a potential role of NBL1 in corneal wound healing and scar formation in humans. 

**Figure 2:**
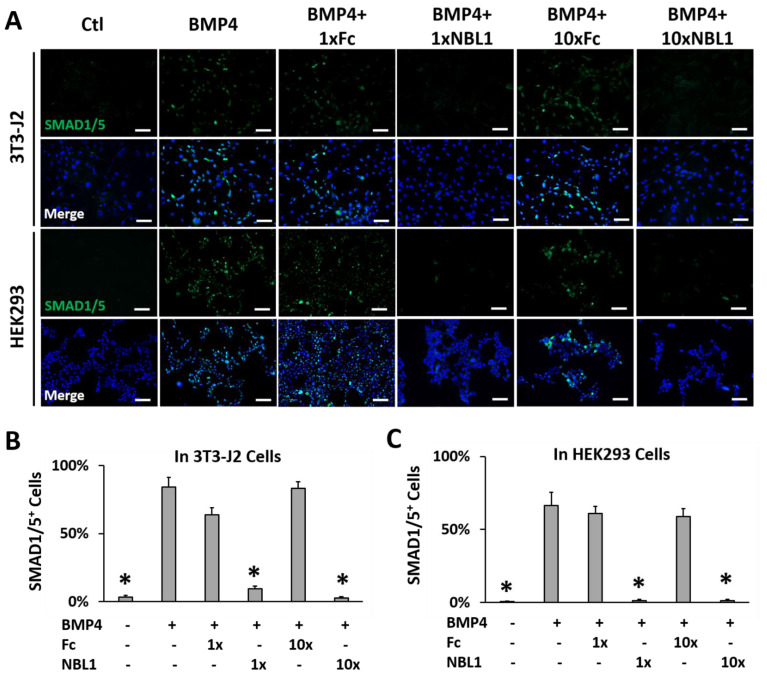
The purchased NBL1-Fc chimeric protein was biologically active as a BMP antagonist in mouse 3T3-J2 cells and human HEK293 cells. Both cell lines were serum starved for 1 h followed by the treatment indicated in figure: ctl (vehicle only), BMP4 (50 ng/mL), BMP4 + 1 × NBL1 (3 µg/mL), BMP + 1 × Fc (same molar concentration as 1 × NBL1), BMP4 + 10 × NBL1 (30 µg/mL), BMP + 10 × Fc (same molar concentration as 10 × NBL1) for 4 h before examination by immunocytochemistry (ICC). (**A**) Representative pictures of these ICC data in 3T3-J2 cells and HEK293 cells. (**B**) Quantitation of these ICC data in 3T3-J2 cells. (**C**) Quantitation of these ICC data in HEK293 cells. N = 4. *: *p* < 0.05. Scale bar: 100 µm.

To examine the possible function of NBL1 in corneal wound healing, wounded corneas were treated with NBL1 using a mouse model and a human organ culture model. NBL1 was purchased from R&D Systems, which is human NBL1 fused with a human IgG1 (Fc) tag. The human IgG1 Fc tag and vehicle served as the two control groups in this study. Before the functional study, we confirmed that the purchased NBL1 fusion protein was biologically active in mouse and human cells. BMP4 activated the BMP signaling and induced phospho-Smad1/5 expression and nuclear translocation, which was suppressed by NBL1, indicating that the purchased NBL1 protein was biologically active as a BMP antagonist in both mouse and human cells. Fc showed no effect on BMP4-induced phospho-Smad1/5 expression or nuclear translocation (Figure 2). 

**Figure 3:**
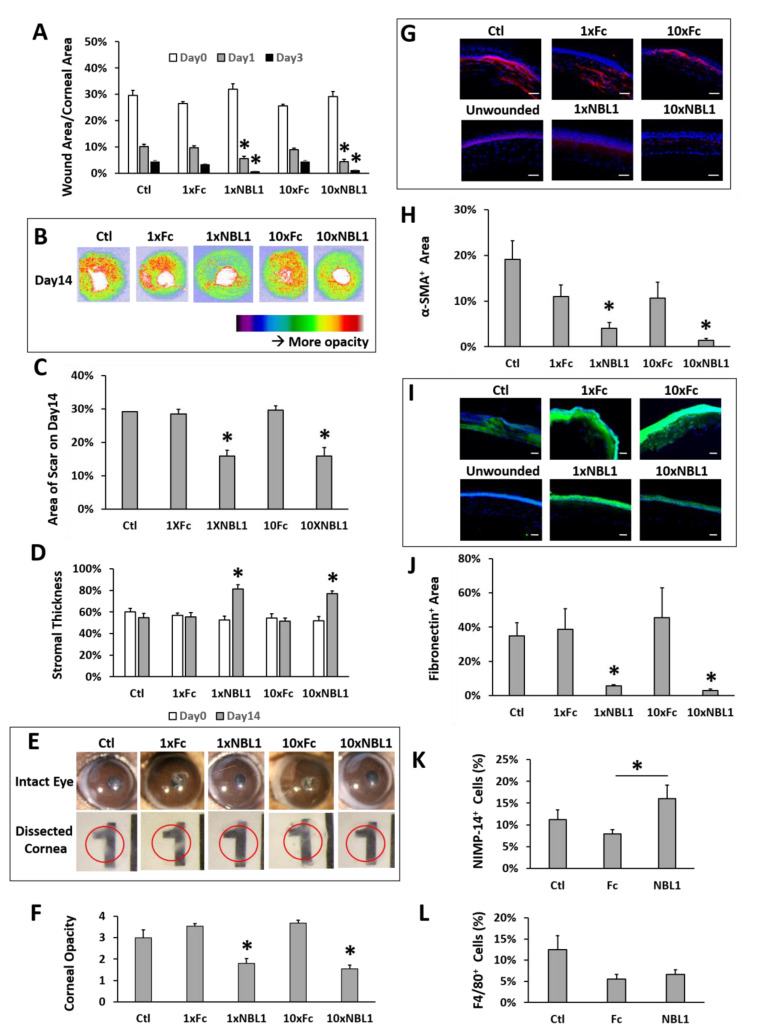
NBL1 treatment reduced corneal scar formation after mechanical wound on mouse corneas. (**A**) NBL1 treatment at both 1× and 10× doses showed a faster wound re-epithelialization revealed by fluorescein staining. (**B**) Representative pictures showing that NBL1 reduced corneal hyper-reflectivity (red and yellow areas, indicating scar) revealed by OCT. (**C**) Quantitative analysis of these OCT data showing that NBL1 reduced the hyper-reflective area indicating the size of the corneal scar. (**D**) Quantitative analysis of these OCT data shows that NBL1 partially restored corneal stromal thickness after a 2-week treatment. (**E**) Representative pictures of intact mouse eyes and dissected mouse corneas show that NBL1 reduced corneal opacity after wounding. The red circle indicates the edge of the dissected cornea, which was placed on a printed “1” to show corneal opacity. (**F**) Quantitative analysis of the corneal opacity based on the double-blind observation and grading from three independent researchers. Corneal opacity: 1/clear, 2/slightly cloudy, 3/very cloudy, and 4/opaque. (**G**) Representative pictures showing that the expression of α-SMA (myofibroblast marker) was significantly reduced in NBL1-treated corneas at both 1× and 10× doses compared with that in the vehicle (Ctl) and the Fc-treated corneas using IHC. (**H**) Quantitative analysis of these IHC data on the percentage of the α-SMA+ area in corneal stroma. (**I**) Representative pictures show that the expression of fibronectin (scar deposition in stroma) was significantly reduced in NBL1-treated corneas compared with that in the vehicle (Ctl) and the Fc-treated corneas using IHC. (**J**) Quantitative analysis of these IHC data on the percentage of the fibronectin+ area in corneal stroma. (**K**) Neutrophil (NIMP-14) infiltration was examined at 24 h after mechanical wounding as the percentage of NIMP-14^+^ area in corneal stroma around the wounding site. (**L**) Macrophage (F4/80) infiltration was examined at 24 h after mechanical wounding as the percentage of F4/80^+^ area in corneal stroma around the wounding site. N = 4–8. *: *p* < 0.05. Scale bar: 50 µm.

After validating the bioactivity of the purchased protein NBL1, we examined its function on corneal wound healing and scar formation in mice. Mouse corneas were wounded by removing corneal epithelium and anterior stroma mechanically using Algerbrush, followed by NBL1 treatment for 2 weeks. To keep the wound consistent among different groups, around 30% of the corneal surface (examined using fluorescein staining) with a depth of 50% of corneal thickness (examined by OCT) was removed. Occasional outliers were excluded from the experiments. Two doses of NBL1 were tested: (1) NBL1 at 3 ng/cornea/injection (named “1 × NBL1”) and (2) NBL1 at 30 ng/cornea/injection (named “10 × NBL1”). Treatment with Fc protein at corresponding molar concentrations (named “1 × Fc” and “10 × Fc”) served as control groups. Treatment with the vehicle served as an additional control (named “Ctl”). During wound healing, wound re-epithelization was monitored using fluorescein staining, which showed the size of the unepithelialized wound. The NBL1-treated groups at both 1× and 10× doses showed a faster wound re-epithelization than Fc groups and vehicle control on Day 1 and Day 3 (Figure 3A and Supplementary Figure S1). At the end of the 14-day treatment, the corneal scar was evaluated using three different methods: OCT (Figure 3B–D), stereomicroscope (Figure 3E,F, Supplementary Figure S2), and protein expression (Figure 3G–J). For Method 1, using in vivo evaluation by OCT, two sets of data were obtained: corneal reflectivity and corneal thickness. Yellow and red colors in OCT images indicate hyperreflectivity caused by opaque scars (Figure 3B); as shown in Supplementary Figure S3, unwounded corneas had minimal yellow and red areas. Quantitative analysis showed that NBL1-treated corneas at both 1× and 10× doses showed smaller hyperreflective scar areas than vehicle control and the corresponding Fc controls (Figure 3C). NBL1 treatment at both 1× and 10× doses showed a partial recovery of stromal thickness from around 52% on Day 0 to 78% on Day 14, while Ctl, 1 × Fc, and 10 × Fc did not show the recovery of stromal thickness (Figure 3D, Supplementary Figure S4). For Method 2 to evaluate the scar using stereomicroscopy, we observed the intact eyes (Figure 3E and Supplementary Figure S2) and the dissected corneas placed above a printed number (number “1” on a ruler) (Figure 3E, red circle indicates the edge of dissected cornea). Corneal opacity was blinded and graded by three researchers with four grades: 1/clear, 2/slightly cloudy, 3/very cloudy, and 4/opaque. Corneas treated with NBL1 at both 1× and 10× doses were less opaque than vehicle control and corresponding Fc controls (Figure 3F). For Method 3 to evaluate the scar by protein expression, the severity of the scar was evaluated based on the presence of abnormal scar myofibroblast cells (α-SMA) and fibronectin expression in the corneal stroma. NBL1 treatment significantly reduced corneal scarring, revealed by reduced α-SMA and fibronectin expression in the corneal stroma (Figure 3G–J). All three methods showed a consistent effect of NBL1 to reduce corneal fibrosis and scar formation after wounding in mice. No difference was observed between the 1× and 10× NBL1 treatments. Whether NBL1 inhibited corneal inflammation, which is a major contributor to tissue fibrosis and scar formation [6,16,17] was studied by examining the immune cell infiltration 1 day after wounding. For neutrophil infiltration, NBL1-treated corneas showed a similar infiltration to the vehicle-treated corneas (“Ctl” in Figure 3) and a higher infiltration than the Fc-treated corneas (Figure 3K and Supplementary Figure S5). For macrophage infiltration, NBL1-treated corneas had a comparable infiltration to the vehicle-treated and the Fc-treated corneas (Figure 3L and Supplementary Figure S5), suggesting that the anti-scar effect of NBL1 was not dependent on immune suppression.

**Figure 4:**
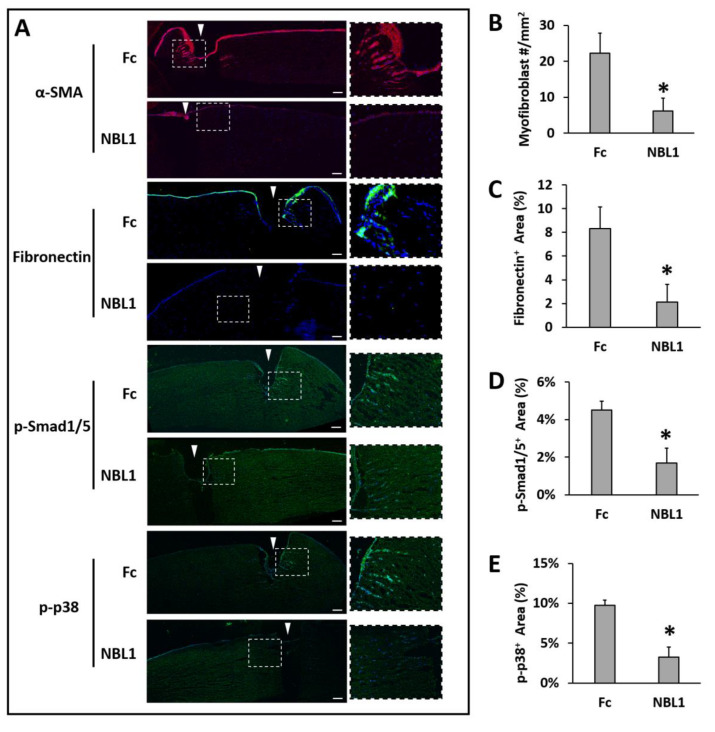
NBL1 inhibited myofibroblast transformation and fibrosis in human corneas upon wounding using an organ culture model. Pairs of human corneas were induced to develop fibrosis by mechanical wounding and incubation in 1 ng/mL TGF-β1 for 1 week. NBL1-treated human corneas showed a significantly reduced number of myofibroblasts (α-SMA) and a significantly reduced scar deposition of fibronectin compared with the Fc-treated corneas (representative pictures in (**A**) and quantitative analysis in (**B**,**C**)). The mechanistic study showed that NBL1 treatment inhibited both BMP canonical (p-Smad1/5) and non-canonical (p-p38) pathways (representative pictures in (**A**) and quantitative analysis in (**D**,**E**)). White arrowheads point to the wound cut by trephine. N = four pairs of human corneas. *: *p* < 0.05. Scale bar: 100 µm.

After we confirmed the anti-scarring effect of NBL1 on mouse corneas, we further examined its effect on human corneas using an organ culture model [14,15]. Pairs of human corneas were cut with a trephine and incubated in a medium containing TGF-β1 to induce fibrosis during wound healing. The left corneas were treated with Fc serving as a control, and the right corneas were treated with NBL1 at 0.3 µg/mL. The control corneas had an obvious myofibroblast formation and fibronectin deposition, especially close to the wounding site, while the corneas treated with NBL1 had significantly reduced myofibroblast cells and fibronectin deposition (Figure 4A–C). We further examined the molecular mechanism of NBL1 to reduce corneal fibrosis under TGF-β1 stimulation. Previous studies showed that activation of the BMP canonical pathway mediated via Smad1/5/8 activation suppressed corneal scar formation in rabbits [18], while the inhibition of p38 (a non-canonical BMP pathway) prevented the transition of human corneal fibroblasts into myofibroblasts under the induction of TGF-β1 [19]. We examined the activation of Smad1/5 and p38 pathways in cultured human corneas and observed that NBL1 treatment significantly inhibited both the canonical (Smad1/5) and non-canonical (p38) BMP pathways (Figure 4A,D,E), which was consistent with its function as a BMP antagonist [20,21,22]. 

## 4. Discussion

Based on our scRNAseq data, NBL1 was highly expressed by corneal stromal cells upon epithelial wounding, which led to scarless wound healing in mouse eyes. Further studies in human corneas confirmed the increased expression of NBL1 in human corneas upon wounding. NBL1 was mainly secreted by activated corneal fibroblasts and myofibroblasts close to the wound. Thus, we hypothesized that NBL1 may play a role in corneal wound healing. To test our hypothesis, mouse corneas were mechanically wounded and treated with the NBL1-Fc fusion protein (NBL1), Fc protein (control), or vehicle (control), respectively. In this experiment, it was crucial to keep a consistent wounding among different groups because the wound size and depth directly affect the severity of the scar [16,23]. We kept the corneal wound at around 25–35% of the corneal surface (examined by fluorescein staining) at a depth of 50–60% of corneal stromal thickness (examined by OCT) consistently among the groups and excluded the outliers from the experiment. The treatment of NBL1 facilitated wound re-epithelialization and significantly reduced corneal scar formation in wounded mouse eyes. To further study the anti-scar effect of NBL1 on human corneas, we performed a test on human corneas in culture. Four pairs of human corneas (death to experiment was completed within 4 days to maximize the responsiveness of the cells to the external stimuli/signals) were obtained from the eye bank. The OS corneas were treated with Fc, and the OD corneas were treated with NBL1. The comparisons between the NBL1 and the Fc (control) groups using a paired sample *t*-test revealed that NBL1 treatment led to a significantly decreased tissue fibrosis after wounding in cultured human corneas, which was consistent with these mouse data. 

Our data suggest some possible mechanisms on how NBL1 inhibited corneal fibrosis and scarring after wounding. The first possible mechanism is that NBL1 may reduce scar formation via facilitating wound re-epithelialization. Previous reports showed that a timely wound re-epithelization is crucial for scarless wound healing, and a persistent epithelial defect can lead to a corneal scar [24]. Our data showed that NBL1 treatment led to a faster wound re-epithelization in mice, which might contribute to its anti-scar effect. However, it should not be the only mechanism because the anti-fibrotic effect was also observed in cultured human corneas, in which the corneas were wounded by a Trephine cut and the wound re-epithelialization was minimal. The second possible mechanism is that NBL1 may reduce scar formation via inhibiting myofibroblast transformation after wounding, which is a key player in tissue fibrosis and scar formation. NBL1 treatment reduced the expression of α-SMA (myofibroblast marker) in wounded mouse corneas in vivo and in wounded human corneas ex vivo. Especially based on data from the ex vivo experiment where the wound re-epithelialization was minimal, and the systemic effect was not present, the reduced number of myofibroblasts in the wounded human corneas with NBL1 treatment suggests the inhibitory effect of NBL1 on myofibroblast transformation which is independent of wound re-epithelialization or systemic inflammation. In addition, NBL1 treatment did not suppress the immune cell infiltration after wounding in mice, which is another evidence showing that the anti-scar effect of NBL1 was not dependent on immune suppression. 

We further examined the molecular mechanism of how NBL1 inhibited the myofibroblast transformation in wounded human corneas. NBL1 is the founding member of the DAN family, which binds to BMPs and inhibits the BMP signaling pathway. Within the DAN family, NBL1 is a moderate BMP antagonist, neither as strong as PRDC/Gremlin/Coco nor as weak as SOST/USAG-1 [21]. Based on our scRNAseq data, NBL1 is the only DAN family member that had an obvious mRNA expression in corneal stromal cells with or without wounding, while other members were minimally expressed (Figure 1A), suggesting that NBL1 is a major player within the DAN family in corneal stromal wound healing. The role of BMPs in tissue fibrosis could be inhibition, activation, or no effect, which depends on the tissue type and specific pathological conditions [25,26,27,28,29,30]. It is possible that NBL1 may inhibit corneal fibrosis by antagonizing the pro-fibrotic BMP pathways in the cornea. Previous reports show different functions of BMP signaling pathways in regulating corneal fibrosis, of which the canonical BMP pathway mediated via Smad1/5/8 is anti-fibrotic [18], and the non-canonical BMP pathway mediated via p38 is pro-fibrotic [19]. Data from IHC showed that NBL1 inhibited both the canonical (Smad1/5) and non-canonical (p38) BMP pathways (Figure 4), which was consistent with its function as a BMP antagonist [20,21,22]. How NBL1 exerts the anti-fibrotic effect on the cornea is still unknown because both the anti- and pro-fibrotic pathways were inhibited upon NBL1 treatment. We hypothesize that the p38 pathway may dominate the Smad pathway during corneal fibrosis, thus leading to the anti-fibrotic effect of NBL1 when both pathways are inhibited. This hypothesis is supported by a previous report, in which the inhibition of the p38 pathway inhibited fibrosis in human corneal fibroblasts, while the inhibition of the Smad pathway had little effect on fibrosis [19], suggesting the dominating effect of the p38 pathway during corneal fibrosis. 

## 5. Patents

Chi Hao Tsai and Hua Mei are the inventors of a patent application related to the anti-fibrotic effect of NBL1 (U.S. Provisional Patent Application No. 63/239,678: “METHODS AND COMPOSITIONS FOR INHIBITING AND REVERSING CORNEAL SCARRING”). The other authors have no conflict of interest to declare.

## Data Availability

Not applicable.

## Supplementary Materials

The following supporting information can be downloaded at: https://www.mdpi.com/article/10.3390/biom13111570/s1, Figure S1: Representative pictures showing that NBL1 treatment at both 1x and 10x doses facilitated wound re-epithelialization revealed by fluorescein staining; Figure S2: Representative pictures showing that a 2-week treatment with NBL1 at both 1x and 10x doses reduced corneal scar formation; Figure S3: Analysis of the OCT data from 8 unwounded mouse corneas showed a minimal hyperreflective (yellow and red) area; Figure S4: Representative pictures showing the measurement of stromal thickness at the wounded area (red lines) and at the unwounded area (yellow lines) on mouse corneas on Day0 and Day14 with NBL1 treatment; Figure S5: Representative pictures showing immune cell infiltration in the wounded mouse corneas at 24 hours after mechanical wounding.
