# Supplementary material for: NBL1 Reduces Corneal Fibrosis and Scar Formation after Wounding
Source: Biomolecules. 2023 Oct 24;13(11):1570. doi: 10.3390/biom13111570 (PMC10669476; doi:10.3390/biom13111570)
Supplement: Supplementary file 1 [file biomolecules-13-01570-s001.zip › biomolecules-2556930-supplementary.pdf]

Supplementary Materials:

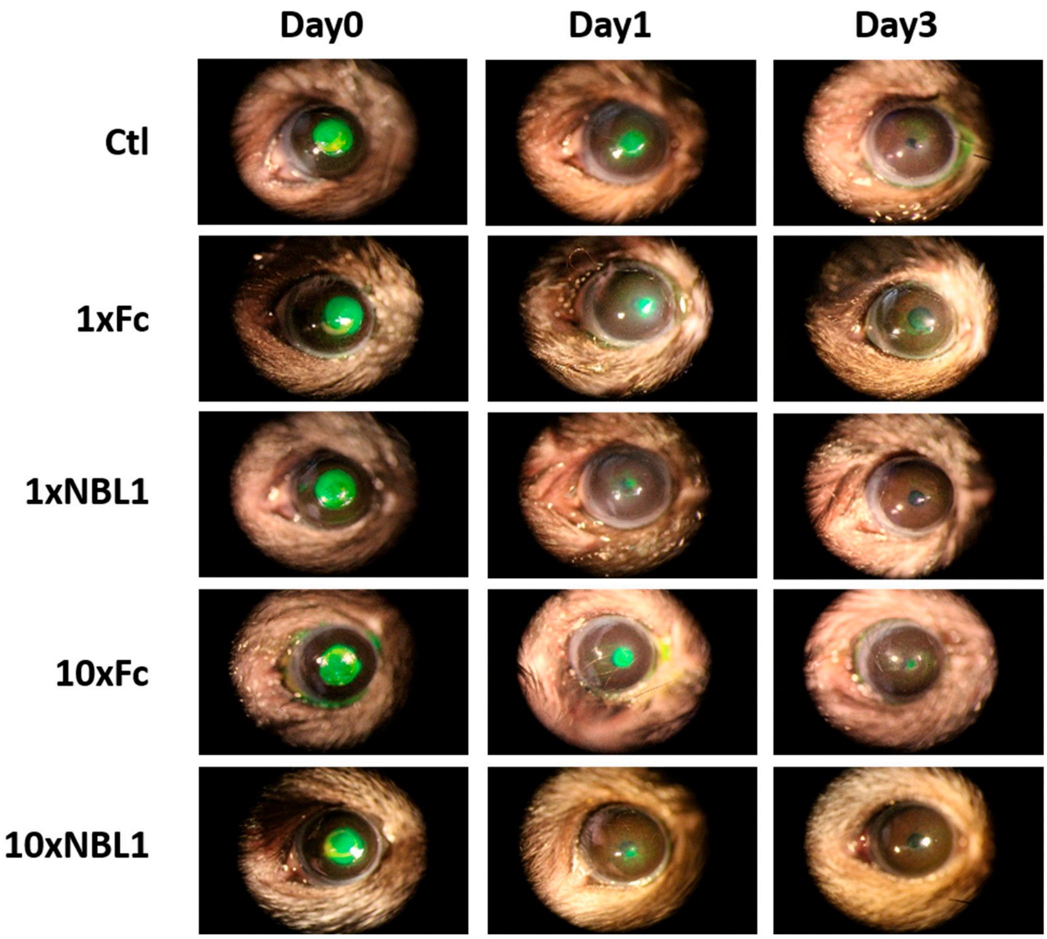

Supplementary Figure S1: Representative pictures showing that NBL1 treatment at both 1x and 10x doses facilitated wound re-epithelialization revealed by fluorescein staining.

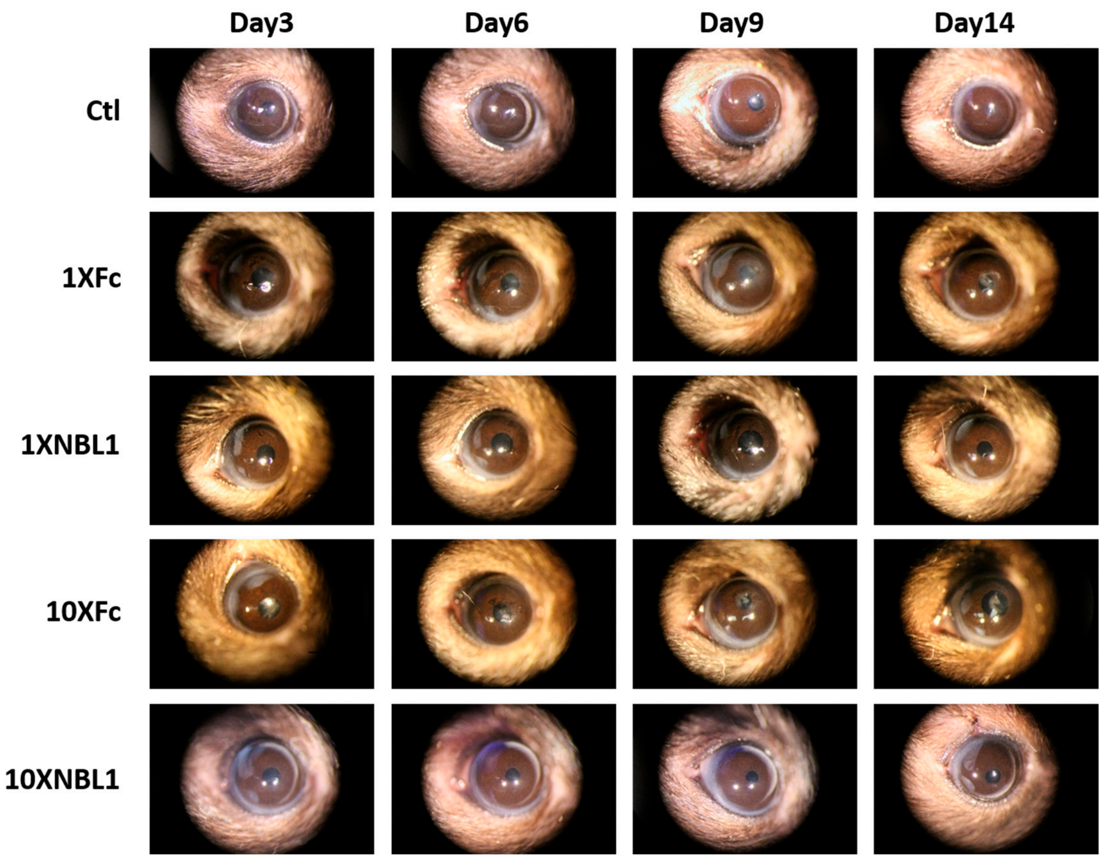

Supplementary Figure S2: Representative pictures showing that a 2-week treatment with NBL1 at both 1x and 10x doses reduced corneal scar formation.

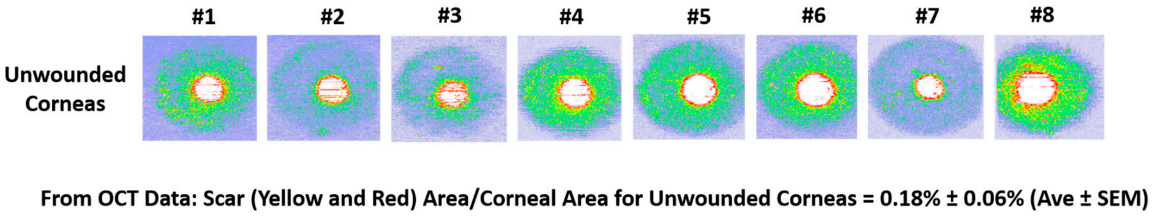

Supplementary Figure S3: Analysis of the OCT data from 8 unwounded mouse corneas showed a minimal hyperreflective (yellow and red) area.

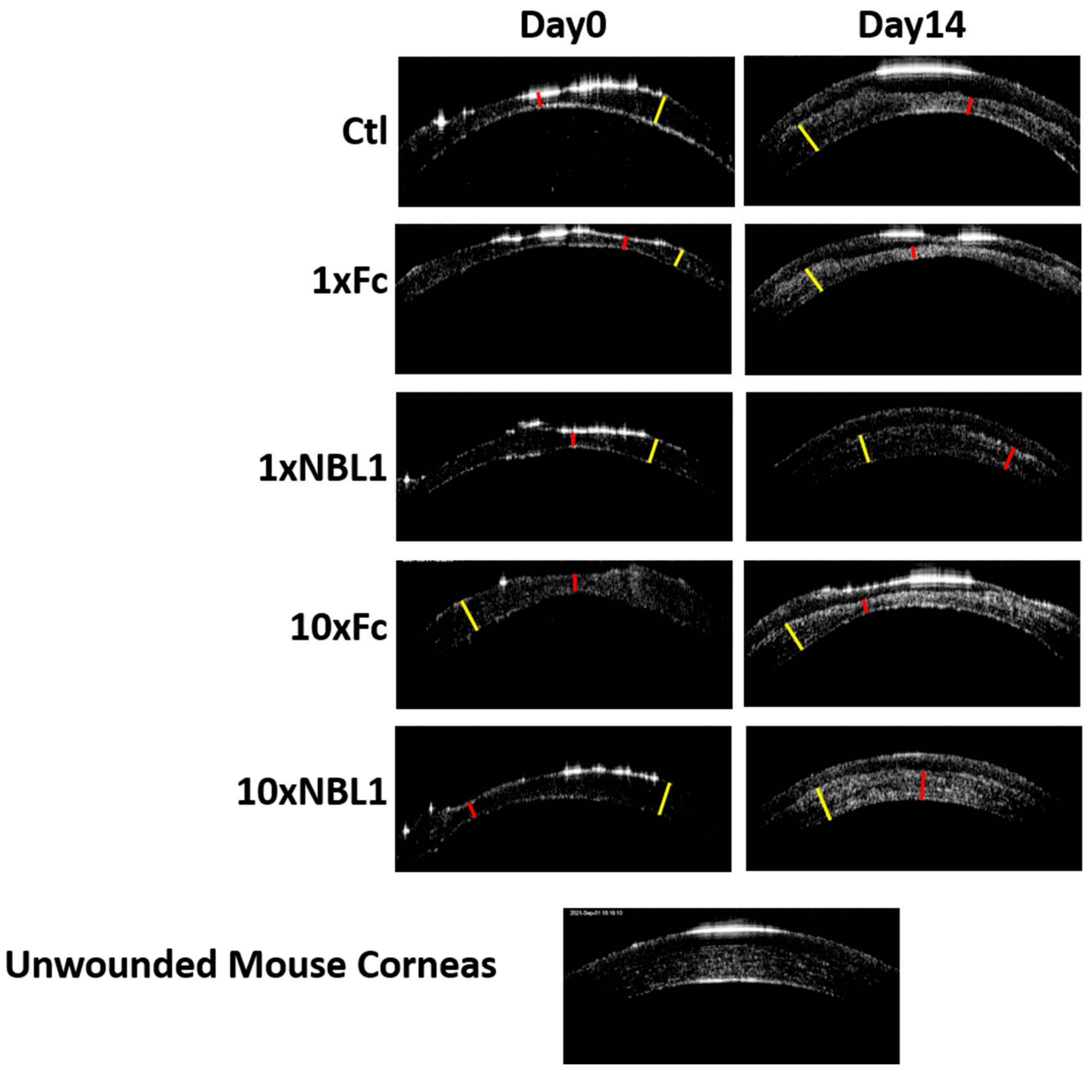

Supplementary Figure S4: Representative pictures showing the measurement of stromal thickness at the wounded area (red lines) and at the unwounded area (yellow lines) on mouse corneas on Day0 and Day14 with NBL1 treatment. OCT data on Day0 were obtained immediately after wounding using the rotating burr which damaged the corneal structure and made the stromal layer indistinguishable on the OCT data, thus the full corneal thickness was measured instead of the stromal thickness on Day0.

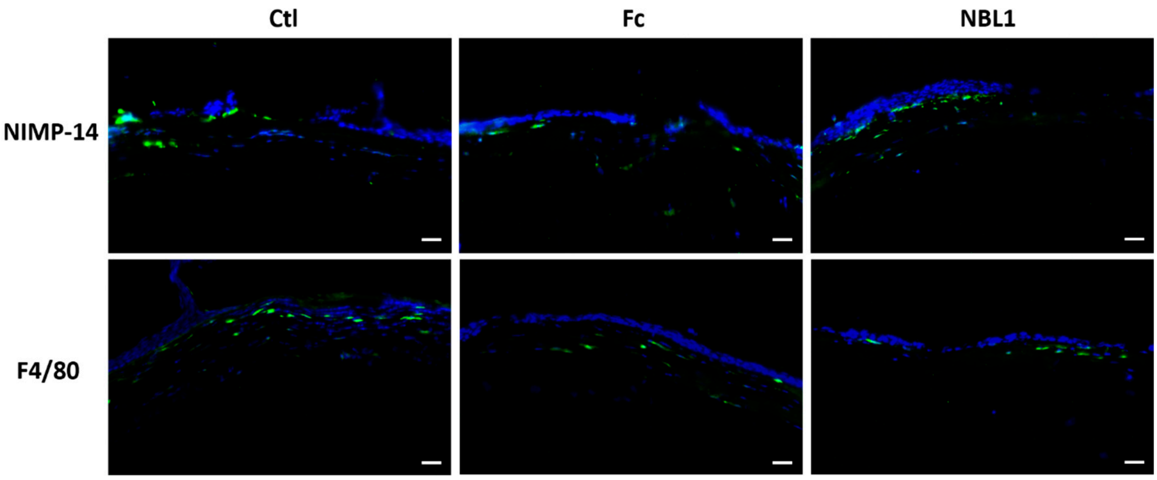

Supplementary Figure S5: Representative pictures showing immune cell infiltration in the wounded mouse corneas at 24 hours after mechanical wounding. Neutrophil (NIMP-14^{+}) and macrophage (F4/80^{+}) infiltration was examined on mouse corneas at the wounding area. Scale bar: 50μm.
